# How Do We Treat Children with Anterior Cutaneous Nerve Entrapment Syndrome and Is the Biopsychosocial Model Also Being Applied? A Scoping Review

**DOI:** 10.1155/2024/6813025

**Published:** 2024-01-29

**Authors:** Anke P. C. Top, Thomas G. de Leeuw, Wichor M. Bramer, Bernadette C. M. de Mol, Frank J. P. M. Huygen, Maaike Dirckx

**Affiliations:** ^1^Department of Anesthesiology, Amsterdam UMC, University Hospital Amsterdam, P.O. Box 22660, 1100 DD, Amsterdam, Netherlands; ^2^Department of Anesthesiology, Erasmus MC-Sophia Children's Hospital, Erasmus University Medical Center, P.O. Box 2040, 3000 CA, Rotterdam, Netherlands; ^3^Center for Pain Medicine, Erasmus MC, Erasmus University Medical Center, P.O. Box 2040, 3000 CA, Rotterdam, Netherlands; ^4^Medical Library, Erasmus MC, Erasmus University Medical Center, P.O. Box 2040, 3000 CA, Rotterdam, Netherlands

## Abstract

**Background:**

Evidence-based guidelines for managing anterior cutaneous nerve entrapment syndrome (ACNES) in children are absent. The primary aim of this review was to scrutinize the evidence supporting currently used treatment interventions. In accordance with the World Health Organization (WHO) guidelines for managing chronic pain in children, these patients and their families and caregivers should be treated within the context of the biopsychosocial model; pain should not be treated purely as a biomedical problem. Therefore, our second aim was to evaluate whether these interventions are applied within the context of the biopsychosocial model, utilizing an inter- or multidisciplinary approach.

**Materials and Methods:**

A scoping review of the literature was conducted to explore treatment strategies for ACNES in children. To ensure a comprehensive overview of published literature on this topic, the search was not restricted based on study type. Two reviewers independently assessed titles and abstracts. After excluding records unrelated to children, full texts were screened for inclusion. Any discrepancies in judgement were resolved through discussion with a third reviewer.

**Results:**

Out of 35 relevant titles, 22 were included in this review. Only 4 articles provided information on long-term outcomes. The overall quality of the review was deemed low. The majority of reports did not address treatment or education within the psychological and social domains. A structural qualitative analysis was not feasible due to the substantial heterogeneity of the data.

**Conclusion:**

The evidence supporting current treatment strategies in children with ACNES is of low quality. More research is needed to establish an evidence-based treatment algorithm for patients with this challenging pain problem. In line with the WHO recommendation, greater emphasis should be placed on a biopsychosocial approach. The ultimate goal should be the development of a generic treatment algorithm outlining an approach to ACNES applicable to all professionals involved.

## 1. Introduction

Chronic recurrent abdominal pain is a prevalent issue in the paediatric population, presenting a wide-ranging differential diagnosis. Only 5–10% of these patients exhibit an underlying physical cause [1]. When no underlying cause is identified, the condition is termed functional abdominal pain. Functional abdominal pain disorders are very common disorders in children, affecting 25% of all infants and children [2].

Anterior cutaneous nerve entrapment syndrome (ACNES) is a pain syndrome characterized by localized abdominal pain resulting from the entrapment of the terminal branches of the intercostal nerves penetrating the muscle sheet of the rectus abdominis. In cases of unilateral chronic or recurrent abdominal pain without an organic explanation, consideration of this diagnosis is warranted, based on specific criteria [3]. Although ACNES was initially described in 1926 by Carnett and Bates [4], it is only in the last decade that this syndrome has been increasingly recognized in children. A notable proportion of adolescents with abdominal pain can now be diagnosed with ACNES [5].

The body of literature describing interventions for the treatment of ACNES, including in children, is expanding rapidly. Various treatment strategies are available, encompassing conservative approaches, invasive treatments involving injections of local anesthetics, and surgical procedures to release the entrapped nerve. Markus et al. conducted a review on the efficacy of neurectomy and trigger point injections in children [6]. Other authors have presented various techniques for peripheral nerve blocks, such as transversus abdominal plane (TAP) block [7–10]. Despite the multitude of patients undergoing treatment for ACNES, there is a notable absence of evidence-based guidelines on how to manage these cases. Most previous publications focus on interventions involving injections and/or surgery. While some reports show promise, the critical question remains: are these treatments superior to conservative approaches and are they applied within the appropriate context?

Chronic pain in children represents a significant health concern that often disrupts daily life, leading to school absenteeism and diminished participation in social activities, thereby impacting their social and emotional development [11]. The treatment of chronic pain in children is complex and often challenging. In addition to addressing underlying organ-specific issues, the central focus in treating abdominal pain involves the rehabilitation of normal daily life. This includes restoring regular meal times, promoting daily exercise and physical activity, and fostering healthy sleep patterns [11]. Extensive evidence supports a multimodal approach in addressing chronic pain in children [11–13]. The development of chronic pain in children involves biological, psychological, genetic, and social factors, all of which should be considered in the diagnosis and treatment of chronic pain [11, 14, 15]. In accordance with the World Health Organization (WHO) guidelines for managing chronic pain in children, these patients and their families and caregivers should be treated within the context of the biopsychosocial model; pain should not be treated exclusively as a purely biomedical problem [16].

With this review, our objectives were to address the following questions:

1. What is the evidence supporting currently used treatment interventions for ACNES in children?
2. Are these interventions applied within the context of the biopsychosocial model, utilizing an inter- or multidisciplinary approach?

## 2. Materials and Methods

The methods in this scoping review are described based on the Preferred Reporting Items for Systematic reviews and Meta-Analyses (PRISMA) checklist [17] and the PRISMA-S extension to the PRISMA Statement for Reporting Literature Searches in Systematic Reviews [18]. An exhaustive search strategy was developed by an experienced information specialist (WB) in consultation with one of the authors (TdL). The search was initially formulated in https://Embase.com, optimized for sensitivity, and then adapted for use in other databases following the approach outlined by Bramer et al. [19]. The search was conducted in the databases https://Embase.com (date of inception 1971), Medline ALL via Ovid (1946 to daily update), and Web of Science Core Collection (Science Citation Index Expanded (1975 to present); Social Sciences Citation Index (1975 to present); Arts and Humanities Citation Index (1975 to present); Conference Proceedings Citation Index-Science (1990 to present); Conference Proceedings Citation Index-Social Science and Humanities (1990 to present) and Emerging Sources Citation Index (2015 to present)); and the Cochrane Central Register of Controlled Trials via Wiley (1992 to present). The search was last updated on 18 January 2023.

The search encompassed terms for (1) anterior cutaneous or lateral cutaneous nerve entrapment and (2) children or pediatrics. Articles not published in English, Dutch, or German were excluded from the search results across all databases. Study registries were not consulted, but Cochrane CENTRAL retrieved the contents of https://ClinicalTrials.gov and the World Health Organization's International Clinical Trials Registry Platform. Full search strategies for all databases are available in the Supplementary Materials (Appendix 1). To provide a comprehensive overview of the published literature on this topic, we did not restrict the search based on study type.

Two reviewers (AT and MD) independently screened titles and abstracts. After excluding records unrelated to children, full texts were reviewed for inclusion. Any discrepancies in judgement were resolved through discussion with a third reviewer (BdM).

## 3. Results

**Figure 1:**
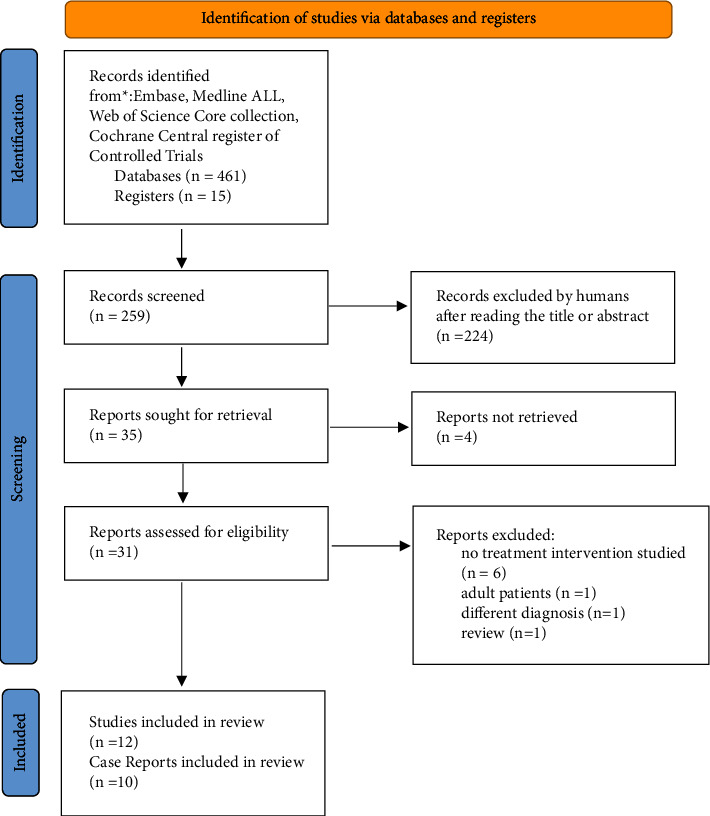
Flowchart with search strategy and study inclusion.

With this search, we identified 476 reports (see Figure 1). After removing 217 duplicates, we screened abstracts and titles, discarding 224 as these pertained to adults. Four reports were excluded due to unavailability of full text. Upon reading of the articles, one more was excluded because it also involved adult patients. Six reports were excluded as they did not study any intervention or treatment and one because the patient had a different diagnosis. In total, we reviewed 23 publications. A systematic review encompassing six studies was identified. However, since these six studies were already included in this review, the additional review was not incorporated into the data [6]. Another report initially appeared to be a review but was, in fact, a prospective observational study assessing the predictive value of a questionnaire [20]. A structural qualitative analysis was not feasible due to the strong heterogeneity of the data.

Five case series (300 patients) and nine case reports (19 patients) reported on injections with local anesthetics. Additionally, five case series (108 patients) and two case reports (2 patients) reported on surgical procedures, and one study detailed outcomes after both injections and surgical interventions (12 patients). Only one article described the results of conservative noninvasive treatment [21]. Among the 11 case series, six originate from the same surgical research group, accounting for 74% of all patients [5, 20, 22–25]. Furthermore, some articles may have overlapping patient populations [7, 22, 23, 26].

### 3.1. Results for Injections with Local Anesthetics

Local anesthetics were injected using different techniques: rectus sheath block, TAP block, or trigger point infiltration, with or without the aid of ultrasound. No comparative studies have been conducted on the technique, the use of ultrasound, or the addition of steroids. Most studies utilized local anesthetics combined with steroids. The median number of injections ranged from 1 to 2, administered at variable time intervals. Reported success rates ranged significantly from 38% to 100% (see Table 1).

### 3.2. Results for Surgical Intervention

The predominantly reported surgical intervention was primarily anterior neurectomy, demonstrating a success rate ranging from 57% to 100%, with a reoperation rate of 25% [5, 22, 25–27]. Additionally, one report described laparoscopic resection of the proximal nerve segment following an unsuccessful anterior neurectomy [27].

Details regarding preoperative treatments were not always clear in these publications. Occasionally, patients received paracetamol, nonsteroidal anti-inflammatory drugs, opioids, and/or antineuropathic medication prior to consultation with the surgeon, but information about medication was not consistently available. All authors report that injections of local anesthetics were administered prior to surgery, either though ultrasound-guided TAP block, rectus sheath block, or trigger point infiltration. The duration of pain symptoms before surgery varied from 6 to 15 (8–29) months across cases (see Table 2).

### 3.3. Results for Conservative Treatment

One study, by García et al. [21], reported on conservative treatment for ACNES. Seven of the twenty patients (35%) reported improvement after oral medication, while one patient reported spontaneous improvement. Five patients received injections. Two of these five were referred to surgery, but pain was resolved before surgical intervention was done.

### 3.4. Results concerning Psychological or Social Factors

Among the 22 studies reviewed, three addressed the social impact of pain, specifically in terms of school absenteeism or limitations in sports activities, affecting 90% of the patients. Five of the 22 studies reported psychiatric or psychological issues in 30% of patients. None of the studies provided commentary about psychologic treatment or physiotherapy.

## 4. Discussion

This literature search aimed to evaluate the evidence regarding treatment interventions for ACNES in children. Surprisingly, the majority of publications focused exclusively on invasive interventions, all of which were observational studies lacking a control group. Consequently, the overall value of evidence is deemed low. Furthermore, hardly any of these publications addressed biopsychosocial factors or treatment options from a biopsychosocial perspective.

Treatment strategies should be tailored to individual patient needs, considering the presence of psychological comorbidities. Nonpharmacological measures, such as cognitive behavior therapy, hypnotherapy, and guided imaginary, have shown efficacy in managing chronic abdominal pain in children and could be viable options [28–30].

Pain education is also deemed crucial in this patient category [29]. The incidence of chronic pain in children is increasing [11], and the body of evidence supporting the value of nonpharmacologic treatments is expanding [31]. This review specifically explores the published treatment options and evaluates their merit.

While the literature on the treatment of ACNES in children is expanding, it predominantly consists of case reports. The methodologic quality of these publications tends to be low. Despite another review claiming level II evidence, we disagree, as there were no randomized controlled trials or meta-analyses and a lack of case-control studies [6]. All six studies were observational nonblinded studies, thus prone to bias.

The existing literature predominantly reports two treatment strategies for managing ACNES in children: injections with local anesthetic (with or without steroids) and surgical procedures. All but one of the relevant publications concern invasive treatments. The only exception is a publication reporting the outcome of conservative treatment in 20 patients, of whom 5 required injections and ultimately none of them needed surgical intervention [21]. This specific study demonstrated that conservative strategies can also be effective.

Most of the publications on injection techniques pertained to either rectus sheath block or trigger point infiltration. The reported success rates ranged from 38% to 100%. Although Bairdain et al. [7] primarily aimed to report surgical outcome, they revealed that all 48 patients received repeated TAP blocks before surgery. Nine of those eventually required surgery, indicating a success rate of 81% with TAP block. While the article did not specifically address whether alternative treatments were pursued or needed, the findings suggest that surgery should only be considered after exhausting other treatment modalities. As of now, no studies have been published comparing the effectiveness of TAP block to other techniques in the treatment of ACNES.

An RCT in adults with ACNES had demonstrated the superiority of lidocaine over saline in providing immediate pain relief after trigger point injection [32]. Reviewing their own previous (mainly observational) ten studies encompassing a total of 834 patients, the same research group estimated that ten percent of the patients experienced sustained pain relief following a single lidocaine injection [33]. While local injections often incorporate steroids, their efficacy in the context of chronic pain remains a topic of debate. A study involving adults in a postoperative setting evidently demonstrated the value of adding steroids to a peripheral nerve block [34]. For chronic pain, however, this is debatable. A study by Mol et al. in adults indicated no discernible benefit of adding steroids to lidocaine injections for ACNES [35].

In another study involving adult patients, Jacobs et al. found that the free hand technique was comparable to the ultrasound-guided technique for trigger point injections [36]. There is a lack of corresponding data in children.

In the context of surgical treatment for children with ACNES, anterior neurectomy emerged as the predominant approach. The duration of preoperative pain symptoms as well as the type of conservative treatment given before surgery exhibited variability. Preoperative interventions lacked standardization, and their specifics were often not clear. Furthermore, a majority of studies featured limited patient cohorts and lacked reporting of long-term follow-up outcomes. Only one retrospective study involving 26 patients reported on extended postoperative outcomes, revealing a 58% success rate [26].

While the biopsychosocial model is recognized as the appropriate framework for addressing pain in children, it is remarkable that a limited number of publications address psychosocial aspects [11]. Among the 22 studies included in this review, merely three explored the social repercussions of pain, such as school absenteeism or limitations in sports activities, affecting 90% of the patients. Psychiatric or psychological issues were reported on five out of the 22 studies, impacting 30% of patients. Van Hoek et al. observed that some patients, who declined surgery after multiple trigger point injections, opted for conservative treatments such as transcutaneous electrical nerve stimulation (TENS), physiotherapy, and psychotherapy [20]. Since the purpose of their study was to determine the predictive value of a questionnaire, they did not describe the outcomes of the different therapeutic options.

The lack of a standardized treatment algorithm for this specific patient category contributes to a varied approach, with no conclusive evidence supporting any particular treatment choice. High-quality research is needed to underpin the development of clear guidelines for the treatment strategy of this elusive diagnosis. Furthermore, it is advisable that interventional procedures be exclusively employed in a multidisciplinary setting [37].

## 5. Conclusions

The quality of the evidence supporting the current practice of treating ACNES in children is low. More research is needed to establish an evidence-based treatment algorithm for patients with this challenging pain problem. In line with the WHO recommendation, greater emphasis should be placed on a biopsychosocial approach. The ultimate goal should be the development of a generic treatment algorithm outlining an approach to ACNES that is applicable to all professionals involved. It is recommended that such an algorithm be implemented within an inter- or multidisciplinary setting, taking into account the biopsychosocial model adapted to the age and social context of the children and their parents.

## Figures and Tables

**Table 1:** Outcome after injections.

Author and year	Study type	Number of patients	Age	Objective	Injection technique	Medication injected	Number of injections	Effect	Follow-up (months)
Aknikh 2014	Case report	3	15 (10–16)		Not described	Local anesthetic + steroid	1	3 (100%) pain-free	>8 (>5–12)

Clara 2022	Retrospective case series	20 (5 receiving injections)	12.9 (11.2–14.1)	To describe the clinical and evolutionary characteristics of patients diagnosed with ACNES and to draw attention to this pathology	Not described	Local anesthetic + steroid	0–2	5/5 (100%) pain-free after injections 8 = 40% pain-free without intervention (7 after medication + 1 spontaneous)	No follow-up

Di Giusto 2018	Case report	1	18		TAP block + trigger point injection	Local anesthetic + clonidine	2 in 1 session	Pain returned much reduced	>12

Ivens 2008	Case report	1	11		Infiltration at nerve exit point abdominal wall	Local anesthetic + steroid			No follow-up

Kifer 2018	Retrospective case series	38	15 (9–17)	To present a treatment modality of ACNES with combined local subfascial anesthetic and corticosteroid injection in a prospectively collected cohort of paediatric patients	US guided rectus sheath trigger point injection	Local anesthetic + steroid	1–7	Total 32 (84%) pain-free	20 (12–32)
								24 (63%) sustained pain-free after 1 injection	
								6 (16%) sustained pain-free after 2 injections	
								2 (5%) sustained pain-free after 3 or > 3 injections	

Lepere 2014	Case report	1	14		TAP block	Local anesthetic + steroid	1	1 pain-free	>12

Minamikawa 2021	Case report	1	15		Rectus sheath block (20 ml)	Local anesthetic	6 (2–4 weeks interval)	1 pain-free	5

Nizamuddin 2014	Case report	3	15 (14–16)		TAP block (with US)	Local anesthetic + steroid	2 (1–3)	2 (66%) pain-free	4–20
								1 lost to follow-up	

Peleg 1999	Case report	1	15		Subcutaneous injection	Local anesthetic + steroid	1	1 pain-free	>24

Siawash 2016	Cross-sectional cohort	12	15 (SD 2)	To investigate the rate of ACNES in a paediatric outpatient cohort with chronic abdominal pain	Trigger point injection	Local anesthetic + steroid	1 (1–4)	5 (42%) pain-free	>4

Siawash 2017 (ped ane)	Prospective case series	85	15 (8–17)	To study outcomes and adverse events of anterior rectus sheath blocks in childhood anterior cutaneous nerve entrapment syndrome	Anterior rectus sheet block	Local anesthetic ± steroid	1–3	32 (38%) pain-free	17 (4–39)

Simpson 2011	Case report	1	13		Bilateral TAP block	Local anesthetic ± steroid	2	1 pain-free	1

Skinner 2007	Case report	7	13 (11–16)		Rectus sheath block	Local anesthetic ± steroid	1–3	5 (71%) pain-free or reduced pain	14 (2–24)
								2 lost to follow-up	

Van Hoek 2022	Prospective case series	145	14.7 (SD 2.3)	Predictive value questionnaire for treatment success	Trigger point injection	Local anesthetic	1–6	36 (25%) pain-free	?

Verma 2018	Case report	1	14		Trigger point injection	Local anesthetic ± steroid	6	Recurrent pain	?

Zganjer 2013	Retrospective case series	12	15 (11–17)	The surgery for abdominal wall pain caused by cutaneous nerve entrapment in children during the past 5 years	Injections	Local anesthetic ± steroid	1–3	7 (58%) pain-free	?

**Table 2:** Outcome after surgical treatment.

Author and year	Study type	Number of patients	Age (years)	Objective	Duration of complaint before surgery (months) Median (range)	Treatment applied before surgery	Surgery technique	Effect	Follow-up (months)	Second surgery	Effect Second surgery
Armstrong 2018	Retrospective case series	26	15 (13.8–17.3)	To evaluate the safety and efficacy of neurectomy for ACNES in children	15 (8–29)	Oral medication	Anterior neurectomy	15 (58%) pain-free	26 (15–36)	4	1 (25%) pain-free
						TAP block with local anesthetic + steroid		11 (42%) recurrent pain			

Bairdain 2015	Retrospective case series	9	14 (10–19)	To report on an entity known as anterior cutaneous nerve entrapment syndrome (ACNES) and its relevance to chronic abdominal pain encountered in children	10 (0.5–60)	TAP block with local anesthetic and/or steroid (1–5)		9 (1 patient still on narcotics)	9 (1–30)		

Omura 2019	Case report	1	16		3	Oral medication injections with local anesthetic	Percutaneous neurectomy	Recurrent pain within 1 month	25	Laparoscopic resection proximal part nerve	Pain-free 2 years

Scheltinga 2011	Prospective case series	6	15 (9–16)	To describe the results of a surgical technique termed “anterior cutaneous neurectomy” in children with refractory ACNES	4	Oral medication? Injections with local anesthetic + steroids	Anterior neurectomy	6 (100%) pain-free	6		

Siawash 2016	Cross-sectional cohort	7	15 (SD 2)	To investigate the rate of ACNES in a paediatric outpatient cohort with chronic abdominal pain	?	Oral medication	Anterior neurectomy	6 (86%) pain-free	4–6 weeks		
						Injections with local anesthetic + steroids					

Siawash 2017 (ped surgery)	Prospective case series	60	15 (SD 2)	To investigate the safety and short-term success rate of anterior neurectomy in a large paediatric population with ACNES	<6 (1–48)	Oral medication? Injections with local anesthetic + steroids	Anterior neurectomy	78%	4–6 weeks		

Yokoyama 2019	Case report	1	16		6	Trigger point injection	Neurectomy		No follow-up		

Zganjer 2013	Retrospective case series	5	15 (11–17)	The surgery for abdominal wall pain caused by cutaneous nerve entrapment in children during the past 5 years		?	Local anesthetic ± steroid	Neurectomy	?	No follow-up	

## Data Availability

The data used to support the findings of the study are available from the corresponding author upon request.

## Supplementary Materials

Supplementary Materials Appendix 1: full search strategies for all databases.

## References

[1] Reust C. E., Williams A. (2018). Recurrent abdominal pain in children. *American Family Physician*.

[2] Thapar N., Benninga M. A., Crowell M. D. (2020). Paediatric functional abdominal pain disorders. *Nature Reviews Disease Primers*.

[3] Siawash M., Van Assen T., Tjon a Ten W. (2019). Abdominal wall pain or irritable bowel syndrome: validation of a pediatric questionnaire. *Journal of Pediatric Gastroenterology and Nutrition*.

[4] Carnett J. B., Bates W. (1933). The treatment of intercostal neuralgia of the abdominal wall. *Annals of Surgery*.

[5] Siawash M., De Jager-Kievit J. W. A., Ten W. T. A., Roumen R. M., Scheltinga M. R. (2016). Prevalence of anterior cutaneous nerve entrapment syndrome in a pediatric population with chronic abdominal pain. *Journal of Pediatric Gastroenterology and Nutrition*.

[6] Markus J., Sibbing I. C., Ket J. C. F., de Jong J. R., de Beer S. A., Gorter R. R. (2021). Treatment strategies for anterior cutaneous nerve entrapment syndrome in children: a systematic review. *Journal of Pediatric Surgery*.

[7] Bairdain S., Dinakar P., Mooney D. P. (2015). Anterior cutaneous nerve entrapment syndrome in children. *Journal of Pediatric Surgery*.

[8] Gulur P., Koury K. M., Lau M. E., Watt L. D., Gulur P. (2014). Use of targeted transversus abdominus plane blocks in pediatric patients with anterior cutaneous nerve entrapment syndrome. *Pain Physician*.

[9] Lepere L., Toelen J. (2014). Chronic abdominal pain in an adolescent: sometimes it is not the content that counts. *Tijdschrift Voor Kindergeneeskunde*.

[10] Simpson D. M., Tyrrell J., De Ruiter J., Campbell F. A. (2011). Use of ultrasound-guided subcostal transversus abdominis plane blocks in a pediatric patient with chronic abdominal wall pain. *Pediatric Anesthesia*.

[11] Landry B. W., Fischer P. R., Driscoll S. W. (2015). Managing chronic pain in children and adolescents: a clinical review. *Physical medicine and rehabilitation*.

[12] Harrison L. E., Pate J. W., Richardson P. A., Ickmans K., Wicksell R. K., Simons L. E. (2019). Best-evidence for the rehabilitation of chronic pain Part 1: pediatric pain. *Journal of Clinical Medicine*.

[13] Liossi C., Johnstone L., Lilley S., Caes L., Williams G., Schoth D. E. (2019). Effectiveness of interdisciplinary interventions in paediatric chronic pain management: a systematic review and subset meta-analysis. *British Journal of Anaesthesia*.

[14] Silva C., Oliveira D., Pestana-Santos M., Portugal F., Capelo P. (2022). Chronic non-cancer pain in adolescents: a narrative review. *Brazilian Journal of Anesthesiology (English Edition)*.

[15] Rivi V., Rigillo G., Toscano Y., Benatti C., Blom J. M. C. (2023). Narrative review of the complex interaction between pain and trauma in children: a focus on biological memory, preclinical data, and epigenetic processes. *Children-Basel*.

[16] Norris S. L., Bamforth I. (2021). *Guidelines on the Management of Chronic Pain in Children*.

[17] Moher D., Liberati A., Tetzlaff J., Altman D. G. (2009). Preferred reporting items for systematic reviews and meta-analyses: the PRISMA statement. *Journal of Clinical Epidemiology*.

[18] Rethlefsen M. L., Kirtley S., Waffenschmidt S. (2021). PRISMA-S: an extension to the PRISMA statement for reporting literature searches in systematic reviews. *Systematic Reviews*.

[19] Bramer W. M., de Jonge G. B., Rethlefsen M. L., Mast F., Kleijnen J. (2018). A systematic approach to searching: an efficient and complete method to develop literature searches. *Journal of the Medical Library Association: JMLA*.

[20] van Hoek P. P., Gorter R. R. R., Janssen L. L., Roumen R. R. M., Scheltinga M. M. R. (2022). The role of a simple questionnaire predicting treatment success in children with ACNES. *Hernia*.

[21] García C. R., Botija A G., Recio L A., Nieto I C., Barrio M A. (2022). Síndrome de ACNES, explorando la pared abdominal como noxa del dolor abdominal. *Andes Pediatr*.

[22] Siawash M., Maatman R., Tjon A Ten W., van Heurn E., Roumen R., Scheltinga M. (2017). Anterior neurectomy in children with a recalcitrant anterior cutaneous nerve entrapment syndrome is safe and successful. *Journal of Pediatric Surgery*.

[23] Siawash M., Mol F., Tjon-A-Ten W. (2017). Anterior rectus sheath blocks in children with abdominal wall pain due to anterior cutaneous nerve entrapment syndrome: a prospective case series of 85 children. *Pediatric Anesthesia*.

[24] Siawash M., Roumen R., Ten W. T. A., van Heurn E., Scheltinga M. (2018). Diagnostic characteristics of anterior cutaneous nerve entrapment syndrome in childhood. *European Journal of Pediatrics*.

[25] Scheltinga M. R., Boelens O. B., Tjon A Ten W. E., Roumen R. M. (2011). Surgery for refractory anterior cutaneous nerve entrapment syndrome (ACNES) in children. *Journal of Pediatric Surgery*.

[26] Armstrong L. B., Dinakar P., Mooney D. P. (2018). Neurectomy for anterior cutaneous nerve entrapment syndrome in children. *Journal of Pediatric Surgery*.

[27] Omura D., Obika M., Iwamuro M. (2019). Anterior cutaneous nerve entrapment syndrome possibly triggered by oral contraceptives. *Internal Medicine*.

[28] Miranda A., Sood M. (2006). Treatment options for chronic abdominal pain in children and adolescents. *Current Treatment Options in Gastroenterology*.

[29] Chakraborty P. S., Daniel R., Navarro F. A. (2023). Non-pharmacologic approaches to treatment of pediatric functional abdominal pain disorders. *Front Pediatr*.

[30] Cordeiro Santos M. L., da Silva Júnior R. T., de Brito B. B. (2022). Non-pharmacological management of pediatric functional abdominal pain disorders: current evidence and future perspectives. *World Journal of Clinical Pediatrics*.

[31] Wren A. A., Ross A. C., D’Souza G. (2019). Multidisciplinary pain management for pediatric patients with acute and chronic pain: a foundational treatment approach when prescribing opioids. *Children*.

[32] Boelens O. B. A., Scheltinga M. R., Houterman S., Roumen R. M. (2012). Randomized clinical trial of trigger point infiltration with lidocaine to diagnose anterior cutaneous nerve entrapment syndrome. *British Journal of Surgery*.

[33] Jacobs M. L. Y. E., Scheltinga M. R. M., Roumen R. M. H. (2021). Persistent pain relief following a single injection of a local anesthetic for neuropathic abdominal wall and groin pain. *Scandinavian Journal of Pain*.

[34] Bailard N. S., Ortiz J., Flores R. A. (2014). Additives to local anesthetics for peripheral nerve blocks: evidence, limitations, and recommendations. *American Journal of Health-System Pharmacy*.

[35] Mol F. M. U., Jansen C. H., Boelens O. B. (2018). Adding steroids to lidocaine in a therapeutic injection regimen for patients with abdominal pain due to anterior cutaneous nerve entrapment syndrome (ACNES): a single blinded randomized clinical trial. *Scandinavian Journal of Pain*.

[36] Jacobs M. L. Y. E., Van Den Dungen-Roelofsen R., Heemskerk J., Scheltinga M. R. M., Roumen R. M. H. (2021). Ultrasound-guided abdominal wall infiltration versus freehand technique in anterior cutaneous nerve entrapment syndrome (ACNES): randomized clinical trial. *British Journal of Surgery Open*.

[37] Vega E., Rivera G., Echevarria G. C., Prylutskyy Z., Perez J., Ingelmo P. (2018). Interventional procedures in children and adolescents with chronic non-cancer pain as part of a multidisciplinary pain treatment program. *Pediatric Anesthesia*.

