# Supplementary material for: How Do We Treat Children with Anterior Cutaneous Nerve Entrapment Syndrome and Is the Biopsychosocial Model Also Being Applied? A Scoping Review
Source: Pain Res Manag. 2024 Jan 29;2024:6813025. doi: 10.1155/2024/6813025 (PMC10843870; doi:10.1155/2024/6813025)
Supplement: Supplementary Materials — Appendix 1: full search strategies for all databases. [file 6813025.f1.docx]

**Appendix 1**

**Detailed search strategy**

The search strategies for Embase and Medline used relevant thesaurus terms from Emtree and Medical Subject Headings (MeSH) respectively. In all databases terms were searched in titles and abstracts of references and the author keywords. The search contained terms for 1) anterior cutaneous or lateral cutaneous nerve entrapment and 2) children or pediatrics. Terms were combined with Boolean operators AND and OR and proximity operators were used to combined terms into phrases. The searches in Embase and Web of Science were limited to exclude conference abstracts published before 2020. In all databases articles not published in English, Dutch or German were excluded from the search results. No study registries were searched, but Cochrane CENTRAL retrieves the contents of ClinicalTrials.gov and World Health Organization's International Clinical Trials Registry Platform. The reference lists of retrieved non-included relevant review articles and of the included references, as well as articles citing these papers have been scanned for relevant references missed by the search. No authors or subject experts were contacted and we did not browse unindexed journals in the field.

The references were imported into EndNote and duplicates were removed by the medical librarian (WMB). Two reviewers (AT and MD) independently screened titles and abstracts. After removal of records that did not concern children, full texts were screened for inclusion. Any discrepancies in the verdict were resolved by discussion with a third reviewer (TdL).

| **Database searched** | **Platform** | **Years of coverage** | **Records** | **Records after duplicates removed** |
| --- | --- | --- | --- | --- |
| Embase | Embase.com | 1971 - Present | 234 | 231 |
| Medline ALL | Ovid | 1946 - Present | 142 | 19 |
| Web of Science Core Collection* | Web of Knowledge | 1975 - Present | 85 | 5 |
| Cochrane Central Register of Controlled Trials | Wiley | 1992 - Present | 15 | 4 |
| **Total** | | | **476** | **259** |

*Science Citation Index Expanded (1975-present) ; Social Sciences Citation Index (1975-present) ; Arts & Humanities Citation Index (1975-present) ; Conference Proceedings Citation Index- Science (1990-present) ; Conference Proceedings Citation Index- Social Science & Humanities (1990-present) ; Emerging Sources Citation Index (2005-present)

**Search strategy used per database**

**Embase.com**

('anterior cutaneous nerve entrapment syndrome'/de OR ('nerve compression'/de AND ('abdominal wall'/de OR 'abdominal wall pain'/de OR 'abdominal pain'/de)) OR (((anterior-cutan* OR lateral-cutan* OR Abdom*) NEAR/6 (nerve-entrap*)) OR (acnes AND nerve-entrap*) OR lacnes):ab,ti) NOT ([conference abstract]/lim AND [2000-2019]/py) AND ([english]/lim OR [dutch]/lim OR [german]/lim)

**Medline ALL Ovid**

((Nerve Compression Syndromes / AND (Abdominal Wall / OR Abdominal Pain /)) OR (((anterior-cutan* OR lateral-cutan* OR Abdom*) ADJ6 (nerve-entrap*)) OR (acnes AND nerve-entrap*) OR lacnes).ab,ti.) AND (english.la. OR dutch.la. OR german.la.)

**Web of Science Core Collection**

TS=(((((anterior-cutan* OR lateral-cutan* OR Abdom*) NEAR/5 (nerve-entrap*)) OR (acnes AND nerve-entrap*) OR lacnes))) AND DT=(article) AND LA=(English OR dutch OR german)

**Cochrane CENTRAL**

((((anterior-cutan* OR lateral-cutan* OR Abdom*) NEAR/6 (nerve-entrap*)) OR (acnes AND nerve-entrap*) OR lacnes):ab,ti)
